# Tax competition, environmental regulation and high-quality economic development: An empirical test based on spatial Durbin model

**DOI:** 10.3389/fpubh.2022.982159

**Published:** 2022-10-28

**Authors:** Xuming Shangguan, Shabir Mohsin Hashmi, Haiya Hu, Wing-Keung Wong

**Affiliations:** ^1^Business School, Xinyang Normal University, Xinyang, China; ^2^One Belt One Road Research Centre, School of Economics and Management, Yancheng Institute of Technology, Yancheng, China; ^3^School of Economics, Guizhou University of Finance and Economics, Guiyang, China; ^4^Department of Finance, Fintech & Blockchain Research Center, Big Data Research Center, Asia University, Taichung, Taiwan; ^5^Department of Medical Research, China Medical University Hospital, Taichung, Taiwan; ^6^Department of Economics and Finance, The Hang Seng University of Hong Kong, Shatin, Hong Kong SAR, China

**Keywords:** high-quality economic development, tax competition, environmental regulation, spatial Durbin model, environmental governance

## Abstract

Studying economic development in China is a very important topic recently because China's economy is moving toward high-quality development and local governments face the dilemma of environmental governance and economic development. To contribute to the literature in this area further, this paper assesses the impact of tax competition and environmental regulation on high-quality economic development through the spatial Durbin model and instrumental variable and by using the data from 278 prefecture-level and above cities from 2007 to 2017 in China. Our empirical analysis shows that tax competition inhibits high-quality economic development and a positive spatial spillover effect, environmental regulation has a significant direct promoting effect on high-quality economic development and a negative spatial spillover effect, and local government tax competition inhibits the promotion effect of environmental regulation on high-quality economic development. Further heterogeneity analysis conducted in our study shows that both the direct and spatial spillover effects of tax competition and environmental regulation on high-quality economic development in large and medium-sized cities are significantly lower than those in small cities. Our empirical analysis infers that since the 18th National Congress of the Communist Party of China, the promotion effect of environmental regulation on high-quality economic development and the synergistic effect with tax competition has become more and more significant. The findings in our paper are useful for both the central government and the local governments in making better decisions for economic development in China as well as in other countries.

## Introduction

Since the reform and opening-up, China's economy has maintained rapid and sustained growth. In 1978, China's GDP was only 367.9 billion Yuan. By 2019, China's GDP is close to 100 trillion Yuan, and it's per capita GDP exceeds 10,000 US dollars. Sustained and rapid economic growth is the fundamental reason for alleviating the contradiction between material and cultural needs and backward social production. However, it has also brought about excessive consumption of resources, ecological destruction, excess production capacity and resource mismatch. The strategic goal of China's development in the new era is to further transform its economy from high-speed growth to high-quality development. Under the new development concept, it is necessary to adhere to the protection of the ecological environment in economic development and the development of the economy in the protection of the ecological environment, which is not only the internal requirement to promote high-quality development, but also the fundamental plan to properly handle the relationship between “two mountains” (1). Therefore, at present and for the period to come, how to effectively promote Hi-Q ED and meet the people's demands for “clear water and green mountains” and “gold and silver mountains” is the focus of economic policy formulation.

Since the 19th CPC, the central government has strengthened its ability to prevent and control environmental risks by strengthening laws and regulations on environmental protection. The quality of the environment has been significantly improved, but environmental pollution still hinders Hi-Q ED in China. The main evaluation index of political promotion of local officials is the level of economic development, so they are more inclined to lower the environmental protection standards and reduce the willingness of regional environmental cooperation governance (2). Especially after the reform of the tax-sharing system, the promotion of local officials is more significantly affected by the level of economic development, forming the “promotion tournaments” with Chinese characteristics that take economic performance as an indicator (3). “Promotion tournaments” lead local government officials to lower tax rates and relax environmental regulation standards in order to attract investment (4). Whether the local government is willing to develop a high-quality economy and how to develop a high-quality economy are not only affected by its willingness to govern the environment, but also affected by its tax policies to promote economic development. Under the new development concept, how the government can promote high-quality economic development through tax competition and environmental governance strategy is worth studying further. And whether there are spatial heterogeneity and regional differences in the impact of tax competition and environmental governance on high-quality economic development. Will tax competition affect high-quality economic development through environmental regulation? Therefore, this paper selects data from 278 cities at the prefecture level and above in China from 2007 to 2017, and systematically examines the impact of both tax competition and environmental governance on high-quality economic development through constructing the spatial Durbin model. Through the research of this paper, it is helpful to solve the confusion of economic development and environmental governance, and provide new solutions for local governments to retain “clear waters and green mountains” and create “gold and silver mountains”.

Compared with the existing research, the marginal of this paper are as follows: (1) Considering the typical fact that the tax and environmental regulations between local governments in China present obvious competition rather than cooperation, the paper systematically investigates the impact of tax and environmental regulations on high-quality economic development for the first time. (2) To overcome the endogenous problem caused by selecting environmental regulation proxy variables from the pollution control results or processes, the paper innovatively constructs the instrumental variables of environmental regulation at the prefecture-level city level. (3) We find that tax competition and environmental regulation have heterogeneous effects on high-quality economic development in different urban scales and periods.

## Literature review

The key to achieving high-quality development is to make innovation the primary driver and improve economic development and environmental protection policies. China's economic growth is in a transitional period of changing its energy, mode and structure. Green governance is an important guarantee for Hi-Q ED in China. However, China's economic structure and driving force are still constrained. Structural adjustment and innovation-driven development are the main driving forces for China's high-quality economic development in the future (5). Cultivating innovators, extending the industrial chain, and developing both the digital economy and new business forms are the green path for the high-quality development of China's economy (6). Although the distribution of economic growth quality in the eastern, central and western regions is unbalanced, it will enter an upward channel in the future (7). The economic quality of China presents a distribution pattern of high in the east, medium in the middle and low in the west; Chinese provinces are divided into three types according to the level of comprehensive level: star type, mediocre type and backward type, which provides a fundamental guarantee for China's comprehensive, coordinated and Hi-Q ED (8).

China's economy has moved from a stage of rapid growth to a stage of high-quality development, and tax reform needs to be deepened to boost high-quality development. Existing studies on Hi-Q ED from tax competition hold that bottom-to-bottom tax competition is an important factor leading to pollution. Top-to-top tax competition is conducive to improving the quality of economic development (9). The actual tax burden difference of enterprises caused by tax competition has a significant impact on the investment decisions of enterprises (10). In order to cater to the motivation of enterprises to move to low-tax areas, the government will provide tax breaks for them, forming a race to the bottom and leading to environmental pollution (11). Preferential tax competition in order to attract international capital inflow to their country promotes economic growth in capital-importing countries, but also causes environmental pollution (12). Tax competition has a significant impact on firms' investment and financing behavior. However, the impact of inter-regional tax competition on investment and financing only exists in non-state-owned enterprises in China, and is not significant in state-owned enterprises (13). Inspired by the political promotion tournament mechanism, tax incentives are tax investment promotion policies generally adopted by the Chinese government. The tax competition among local governments aggravates the ecological deterioration, which is inconsistent with the concept of Hi-Q ED (14). In order to promote economic development, local governments carry out unreasonable and non-compliant tax competition. After economic development reaches a certain level, the promoting effect on economic development gradually turns to the inhibiting effect (15). Tax competition is one of the main means for local governments to compete for liquidity elements in China, and there exist both strategic complementarity and strategic substitution in tax policy. In China, inter-regional tax competition has a significant reverse impact on industrial transfer, and the tax competition of industrial transfer tends to be diversified (16). In order to effectively reduce capital tax and attract floating capital to promote local economic development, the preferential tax policies of local governments improve the environment for attracting investment, but also cause problems such as resource misallocation, environmental pollution and excess capacity (17). Local governments produce tax “depressions”, forming a vicious competition of “bottom-chasing” tax burden, which hinders the integration of regional resources in China (18). Tax efforts exist in strategic imitation competition among regions, and the efficiency of local tax collection and administration is positively correlated with capital flows. In contrast, the efficiency of tax collection and administration in neighborhood regions is negatively correlated with capital flows (19). In non-cooperative competition without tax policy coordination, the asymmetry of inter-regional tax policies will lead to the spatial asymmetry of industrial distribution and economic development occurring in advance and magnifying over time (20). The inter-regional tax competition in China has an obvious spatial correlation. Tax competition inhibits the green development of the local region, but promotes the green development of neighboring regions (21). Based on the above analysis, this paper proposes hypothesis 1:

Hypothesis 1: Tax competition not only inhibits the high-quality development of the local economy, but also has a positive spatial spillover effect due to the externality of tax competition.

Greening is one of the indicators of Hi-Q ED. Most of the existing research mainly investigates the environmental pollution effect of economic development and environmental regulation, and ignores the impact of environmental regulation on the quality of economic development (22). However, since the 18th CPC National Congress, how environmental regulation affects the quality of economic development has become the focus of Chinese scholars' attention. There is a threshold effect on the impact of environmental regulation on the quality of economic development (23). On the whole, environmental regulation has improved the quality of China's economy (24), from a subdivided perspective, environmental regulation has significantly promoted green development, economic efficiency and social welfare, but failed to promote the optimization and upgrading of industrial structure (25). Since China's economic growth is an inverted u-shaped relationship between quality and environmental regulation, China's environmental regulation is still at a turning point on the left side of the intensity, properly strengthening the environmental regulation structure and helping to promote enterprise technology innovation to achieve effective to improve the quality of economic growth, is the important path to realize Hi-Q ED (26). Haze pollution hurts Hi-Q ED, the effect of environmental regulations on inhibiting haze pollution is not significant (27). In order to achieve Hi-Q ED driven by ecological environmental protection, it is necessary to strengthen the leading and forcing role of ecological environmental protection and the rigid constraint role of ecological environmental protection laws and regulations (28). Based on the above analysis, this paper proposes hypothesis 2:

Hypothesis 2: Strengthening environmental regulation has a great effect on the green development of the local economy. Due to the externality of environmental regulation, there is a negative spatial spillover effect on the Hi-Q ED of neighboring areas.

To sum up, abundant results have been achieved in existing studies on the impact of environmental regulation and tax competition on Hi-Q ED. However, the typical facts of Chinese local governments are obvious competition rather than cooperation in economic development and environmental governance. The existing literature has not made a systematic and in-depth investigation on it, and ignored the fact that Hi-Q ED requires coordination among regional governments. China's local tax incentives are in a dilemma of “one tube will die, one put on chaos”, and the policy of “tax paradise” is the dominant strategy only in areas with low governance intensity (29). Tax competition between local governments and environmental control policies influence each other (30). Under the new development concept, to achieve “clear water and green mountains”, local governments not only need to improve the intensity of environmental regulation, but also need to change the concept and mode of regional tax competition. Tax competition is an externality, and pollutants are fluid. Since pollutants generated by getting low-tax competition will cause environmental pollution in neighboring areas, studying the coordinated green development of the regional economy could be beneficial (31). Based on the above analysis, this paper proposes hypothesis 3:

Hypothesis 3: Environmental regulation can lead to green tax competition, and environmental regulation and tax competition have synergistic effects on Hi-Q ED.

## Methods and data

### Model setting

According to the literature review, tax and environmental regulation policies are effective tools for local governments to compete for mobile capital. Local governments will adjust local policies according to the tax policies of neighboring regional governments, and this adjustment behavior has a spatial interaction effect. Moreover, environmental pollution is spatially fluid, and the adjustment of local government environmental regulation policies will not only lead to the change in local environmental pollution, but also lead to the change in pollution degree in neighboring areas. The local environmental protection level will also be affected by the environmental regulation strategies in neighboring areas. Therefore, relevant variables reflecting spatial interaction effects should be introduced into the empirical model to test the effects of tax competition and environmental regulation on Hi-Q ED.

The Moran index I test results of explained variable and core explanatory variable data based on geographical distance spatial weight (W)^1^ are reported in Table 1; it can be seen that the null hypothesis of no spatial autocorrelation is rejected by economic development quality, tax competition and environmental regulation at the significance level of 1%; This suggests that economic development quality has spatial autocorrelation. Moreover, it will be affected by the level of taxation and environmental regulation in neighboring areas. Therefore, the empirical model should include the spatial interaction effect of taxation, environmental regulation and the quality of economic development.

**Table 1:** Moran index I.

**Variables**	**2007**	**2009**	**2011**	**2013**	**2015**	**2017**
ln*tax*	0.066^***^	0.073^***^	0071^***^	0.084^***^	0.078^***^	0.063^***^
ln*ere*	0.114^***^	0.109^***^	0.113^***^	0.106^***^	0.134^***^	0.142^***^
ln*gtfp*	0.176^***^	0.166^***^	0.181^***^	0.166^***^	0.188^***^	0.191^***^

*** represent significance levels of 10, 5 and 1%, respectively.

If the variables in the model have spatial interaction, the traditional econometric model may not be able to analyze them, so the spatial econometric model should be used. The spatial Durbin model includes the spatial interaction effects of both explanatory and explained variables, which can solve the problem that the spatial lag model or spatial error model does not fully investigate the spatial interaction effects (32). At the same time, through LR test and Wald test, it is found that both reject the original assumption that SDM model degenerates into SEM or SAR model), as shown in Table 2, which shows that SDM model is the best compared with SEM model and SAR model.

**Table 2:** Control variables and data sources.

**Variable name**	**Variable measure**	**Unit**	**The data source**
FDI	The proportion of foreign direct investment in GDP	%	China city statistical yearbook and website of national bureau of statistics
Level of financial development	Per capita loan balance of financial institutions at year-end	yuan	Statistical yearbook of Chinese cities
Consumption level	Per capita annual consumption in the city	yuan	Statistical yearbook of Chinese cities
Industrial structure	Proportion of added value of the secondary industry in GDP	%	Statistical yearbook of Chinese cities
Urban greening rate	Green coverage rate in built-up areas	%	China Urban construction statistical yearbook
Information level	Number of internet users in cities	Ten thousands of families	China Urban construction statistical yearbook
Basic traffic	The number of buses per 10,000 people	Car	China Urban construction statistical yearbook
Urban population density	Population per square kilometer of urban area	People per square kilometer	China Urban construction statistical yearbook

In order to mitigate the impact of both heteroscedasticity and skewness on the model estimation results, the model adopts the logarithmic function form (33). The following model (model 1) is first used in this paper:


(1) $$\begin{array}{l} \begin{array}{l} \ln\;gtf{\;p}_{it}=\rho W\ln\;gtf{\;p}_{it}+\beta_{1}\ln\;tax_{it}+\beta_{2}\ln\;ere_{it}\\ \;+\beta_{3}\ln\;tax_{it}*\ln\;ere_{it}\\ \;+\theta_{1}W\ln\;tax_{it}+\theta_{2}W\ln\;ere_{it}+\lambda \ln\;X+\alpha_{i}\\ \;+\gamma_{t}+\nu_{it} \end{array}, \end{array}$$

where *i* represents the city, *t* represents the year, *gtfp* measures the level of Hi-Q ED, *tax* reflects the level of tax competition among local governments, *ere* represents the intensity of environmental regulation, and *X* represents urban characteristics and economy-related control variables. In addition, is the city fixed effect, γ_*t*_is the time fixed effect, ***W*** is the spatial weight, ρ is the spatial autoregressive coefficient, and *v*_*it*_is the error term. Since Model (1) contains spatial interaction terms such as ***W*** ln *gftp*, ***W*** ln *tax* and ***W*** ln *ere*, the regression coefficient obtained from point estimation cannot reflect the corresponding spatial spillover effect. Partial differential estimation can effectively resolve the defects of point estimation in measuring the spatial effect and provide a solid foundation for testing the spatial effect. The specific derivation process is as follows:

Firstly, the spatial Durbin model in general form is transformed into Equation (2):


(2) $$\begin{array}{l} Y={\left(I\text{-}\rho W\right)}^{-1}\left(X\beta +WX\theta \right)+\varepsilon . \end{array}$$

Calculate the partial derivatives of k explanatory variables of Equation (2), and get the expected partial derivatives matrix (3):


(3) $$\begin{array}{l} \left[\frac{\partial E\left(Y\right)}{\partial x_{1k}}•\frac{\partial E\left(Y\right)}{\partial x_{Nk}}\right]=\left[\begin{array}{ccc} \frac{\partial E\left(y_{1}\right)}{\partial x_{1k}} & • & \frac{\partial E\left(y_{1}\right)}{\partial x_{Nk}}\\ • & • & •\\ \frac{\partial E\left(y_{N}\right)}{\partial x_{1k}} & • & \frac{\partial E\left(y_{N}\right)}{\partial x_{Nk}} \end{array}\right]\\ \;={\left(I-\rho W\right)}^{-1}\left[\begin{array}{cccc} \beta_{k} & w_{12}\theta_{k} & • & w_{1N}\theta_{k}\\ w_{21}\theta_{k} & \beta_{k} & • & w_{2N}\theta_{k}\\ • & • & • & •\\ w_{N1}\theta_{k} & w_{N2}\beta_{k} & • & w_{NN}\beta_{k} \end{array}\right]. \end{array}$$

The mean values of diagonal elements in Equation (3) are used to measure the direct effects, the mean values of non-diagonal elements are used to measure the spatial spillover effects (indirect effects), and the sum of the direct effects and spatial spillover effects is the total effect.

### Variables and data

#### Explained variables

Hi-Q ED (*gtfp*). Existing literature fails to reach a consensus on how to measure Hi-Q ED. Some have constructed an index system, while others use per capita GDP. However, green development is not considered, which is inconsistent with the new development concept. In this paper, green total factor productivity is adopted to represent the high-quality development level of the regional economy. The specific measurement process of green total factor productivity refers to the practice of Fare et al. Malmquist-luenberger index was used to calculate urban green total factor productivity (34).

#### Explanatory variables

(1) Tax competition. Tax competition is one of the important means to attract liquidity factors, optimize resource allocation and affect economic development. Although China's tax rate is uniformly controlled by the central government, in order to attract floating capital, local governments can reduce the local effective tax rate by reducing the efficiency of collection and management, financial return, subsidies and lowering land transfer fees in the process of actual operation (35). Existing studies also confirm significant tax competition between local governments in China (36). However there is still no consensus on the indicators of tax competition in the existing literature. The actual tax burden of the region is measured by the proportion of regional enterprise income tax, business tax and value-added tax in the added value of the secondary and tertiary industries (29), at the same time, considering that the means of tax competition between local governments for mobile capital is mainly aimed at the industrial sector, which is also the main object of environmental regulation. And this paper uses the tax revenue per unit of industrial output as the proxy variable of regional tax competition in the empirical study, that is, the reciprocal ratio of total regional industrial tax revenue to total industrial output value is used to represent the degree of tax competition between local governments. According to the tax competition index in this paper, when the tax revenue per unit of industrial output in a region is low, the region has higher tax competition intensity than other regions; otherwise, the tax competition intensity in this region is low. The above data are from China City Statistical Yearbook.

(2) Environmental Regulation (*ere*). Leonie et al. think that the choice of environmental regulation strategies by local governments mainly depends on their willingness to control the environment, and it is more comprehensive and objective to investigate environmental regulation from the pollution control results (37). Concerning the two indicators of industrial smoke (powder) dust removal rate and sulfur dioxide removal rate selected by Shen et al. (22), this paper also increases the comprehensive utilization rate of solid waste and the standard rate of wastewater discharge, forming the comprehensive index (*ere*) of environmental regulation at the urban level required. The specific steps are as follows:

Step 1: Standardize the original value of the pollutant. For the comparability of the data, Equation (4) is constructed to standardize the four individual indexes of the comprehensive index of environmental regulation.


(4) $$\begin{array}{l} scp_{ijt}=\frac{cp_{ijt}-\min\left(cp_{jt}\right)}{\max\left(cp_{jt}\right)-\min\left(cp_{jt}\right)}, \end{array}$$

where *scp*_*ijt*_ is the standardized value of pollutant *j* produced by city *i* in period *t, cp*_*ijt*_ is the original value of pollutant *j* produced by city *i* in period *t*, max (*cp*_*jt*_) represents the maximum amount of pollutant *j* in all cities in period *t*, and min (*cp*_*jt*_) represents the minimum amount of pollutant *j* in all cities in period *t*.

Step 2: Construct a reasonable weight of urban pollutants. Due to the heterogeneity of the proportion of wastewater discharge, solid waste, sulfur dioxide and industrial smoke (powder) production and discharge in different cities, the emission of different pollutants in the same city is also different. In order to ensure that the comprehensive index of environmental regulation can correctly reflect the intensity of urban environmental regulation, the weight formula (5) is constructed in this paper.


(5) $$\begin{array}{l} w_{ijt}=\frac{P_{ijt}}{\sum P_{ijt}}/\frac{Y_{ijt}}{\sum Y_{ijt}}, \end{array}$$

where *p*_*ijt*_ represents the amount of pollutant *j* discharged by city *i* in period *t, Y*_*it*_ represents the GDP of city *i* in period *t*, and *w*_*ijt*_ represents the ratio of the emission of pollutants *j* in city *i* in the period of *t* to the national emission of pollutants *j* in the period of *t* to the GDP of the city *i* in the period of *t* to the national GDP. The logic of using *w*_*ijt*_ as the weight is that if the pollutant *j* emitted by city *i* is relatively high in the period of *t*, the same removal rate means stronger environmental regulation, which needs to be given greater weight.

Step 3: Calculate the comprehensive index of environmental regulation. According to the standardized values and weights of the above four emissions, the environmental regulation intensity of city *i* in period *t* can be calculated by the following equation:


(6) $$\begin{array}{l} ere_{it}=4/\sum_{j}^{4}w_{ijt}scp_{ijt}. \end{array}$$

Among them, *ere*_*it*_ represents the environmental regulation intensity of city *i* in year *t*. The greater *ere*_*it*_ is, the greater the local government's pollution control intensity is, and vice versa. The above data are from China Urban Statistical Yearbook and China Environmental Statistical Yearbook.

#### Control variables

In order to reduce the errors of model regression results caused by missing variables, the Spatial Durbin Model as shown in (1) constructed in this paper is further controlled for economic correlation and urban characteristic variables. The important factor affecting the quality of economic development is the main way of economic development (1, 38). If relevant variables are omitted from the model, the estimation results will be unreliable. The variables of economic development mode controlled in the model include industrial structure (*industry*), level of financial development (*finance*), utilization of foreign capital (*fdi*) and consumption level (*consume*). For the sake of further alleviate the estimation bias caused by missing variables, the control variables in this paper also include urban green rate (*green*), basic traffic (*traffic*), information level (*info*) and urban population density (*popu*). Related control variables and data sources are shown in Table 3. Among them, variables that cannot be directly obtained are calculated based on basic data such as the China Urban Statistical Yearbook, China Urban Construction Statistical Yearbook and the website of the National Bureau of Statistics. With the help of the provincial residents' consumption level index (2007 = 100), the monetary value variable was deflated to eliminate the impact of price changes. The actual utilized foreign capital was converted from US dollars to RMB by the current exchange rate.

**Table 3:** LR and Wald test.

**Test method**	**Weight of adjacent space**		**Spatial weight of geographic distance**	
	**Eigenvalue**	***p***	**Eigenvalue**	***p***
LR-lag test	126.40	0	78.84	0
LR-error test	273.52	0	204.89	0
Wald-lag test	71.43	0	178.56	0
Wald-error test	487.53	0	258.41	0

## Results and discussion

### Basis regression results based on spatial Durbin model

This paper uses the maximum likelihood method to estimate the spatial Durbin model. The estimated results are reported in Table 4.^2^ Compared with Column (1), Column (2) includes the interaction item of tax competition and environmental regulation in the model to investigate the synergistic effect of tax competition and environmental regulation on the quality of economic development. After controlling for variables related to urban and economic characteristics, the results show that the spatial auto-correlation coefficient (ρ) is significantly positive at the significance level of 1%, which is consistent with the Moran index *I* test results above, and supports the theoretical analysis that the quality of economic development has strong spatial auto-correlation. Tax competition has suppressed the promotion of local economic development quality, the main reason is that “promotion tournament” induces tax competition among local governments to compete for the bottom, which reduces the cost of capital flowing to polluting industries, forms resource mismatch, inhibits the willingness and motivation of enterprises to adopt advanced technologies, and is not conducive to Hi-Q ED. The impact of environmental regulation on the quality of local economic development is significantly positive, that is, increasing the intensity of environmental regulation can promote the improvement of urban green total factor productivity. The interaction coefficient between environmental regulation and tax competition is not significant, but it's positive, indicating that the bottom-to-bottom tax competition behavior among local governments inhibits the pushback effect of environmental regulation on Hi-Q ED. The regression coefficient of the spatial effect of tax competition is significantly positive, indicating that local bottom-seeking tax competition will lead to the relocation of polluting enterprises to the local area, which is conducive to the Hi-Q ED of neighboring areas. The regression coefficient of the spatial spillover effect of environmental regulation is significantly negative, indicating that with the increase of environmental regulation intensity, polluting industries will move to the neighboring areas with weak environmental regulation intensity, which is not conducive to the high-quality development of a neighboring local economy.

**Table 4:** The regression results of spatial Durbin model.

	**Core explanatory variable: current period**		**Core explanatory variable: lag one**	
**Variable**	**(1)**	**(2)**	**(3)**	**(4)**
***W***×ln*gtfp*	0.418***	0.425***	0.418***	0.426***
	(0.131)	(0.133)	(0.129)	(0.131)
ln*tax/* L.	−0.062***	−0.071***	−0.051***	−0.066***
	(0.016)	(0.021)	(0.011)	(0.017)
ln*ere/* L.	0.084***	0.088***	0.093***	0.089***
	(0.019)	(0.023)	(0.024)	(0.022)
ln*tax × *ln*ere/* L.		0.560		0.610
		(0.410)		(0.420)
***W***×ln*tax/* L.	0.054*	0.058*	0.068***	0.076****
	(0.029)	(0.032)	(0.021)	(0.025)
***W***×ln*ere/* L.	−0.064**	−0.067***	−0.074**	−0.092***
	(0.031)	(0.023)	(0.036)	(0.031)
ln*green*	0.039	0.061**	0.062	0.084**
	(0.028)	(0.029)	(0.047)	(0.036)
ln*traffic*	0.067*	0.059**	0.065	0.047**
	(0.036)	(0.026)	(0.041)	(0.023)
ln*info*	0.016	0.017*	0.014	0.019*
	(0.014)	(0.010)	(0.013)	(0.011)
ln*popu*	−0.017***	−0.018***	−0.016***	−0.019***
	(0.004)	(0.005)	(0.003)	(0.006)
ln*industry*	−0.019**	−0.024**	−0.017**	−0.016**
	(0.008)	(0.011)	(0.008)	(0.007)
ln*fdi*	−0.036*	−0.039**	−0.043*	−0.047**
	(0.019)	(0.018)	(0.024)	(0.023)
ln*finance*	0.016	0.011	0.039*	0.023
	(0.015)	(0.010)	(0.021)	(0.019)
ln*consume*	0.045	0.043	0.038*	0.039
	(0.038)	(0.036)	(0.021)	(0.024)
Urban fixed effect	yes	yes	yes	yes
Time fixed effect	yes	yes	yes	yes
Number of observations	3,058	3,058	2,780	2,780

*, **, and ***represent the significance level of 10, 5, and 1%, respectively.

The values in brackets are the corresponding robust standard errors, and L. represents a lag of 1 period.

From the coefficient of relevant control variables, basic transportation and urban greening have significantly promoted the Hi-Q ED at the significance level of 5%, information has a positive impact on Hi-Q ED at the significance level of 10%, the effect of Hi-Q ED of population density is negative at the significance level of 1%, and thus improving the urban environment is conducive to Hi-Q ED. At the significance level of 5%, the utilization of foreign capital and industrial structure hurt Hi-Q ED. In contrast, both financial development and consumption have positive but not significant effects on Hi-Q ED. Therefore, it is necessary to further promote the upgrading of industrial structure, improve the quality of foreign investment, and promote the transformation of the economy from extensive development to high-quality development.

In order to avoid the lag effect of environmental regulation and tax competition leading to the unreliability of the model estimation results, the one-period lag of model (1) is used for regression. The estimated results are reported in columns (3) and (4) of Table 4, compared with the results in Column (1) and (2), the negative impact of tax competition on Hi-Q ED and the positive impact and significance of environmental regulation are consistent with the current period, the lag effect has no significant impact on the estimated results, and the discussion will be based on current variables in the following paragraphs.

It is not accurate to use regression coefficient directly to analyze the interaction between variables in the spatial econometric model, and it should use direct effect and spatial spillover effect (indirect effect) to explain. Table 5 reports the direct effect, spatial spillover effect and total effect of local government tax competition and environmental regulation on Hi-Q ED. The direct effect of tax competition on the quality of economic development is significantly negative, while the direct effect of environmental regulation is significantly positive. The quality level of economic development decreases by 0.069% on average when tax competition increases by 1%, while the quality level of economic development increases by 0.081% on average when the intensity of environmental regulation increases by 1%. The spatial spillover effect of tax competition on the economic development quality of neighboring areas is positive, that is, at the significance level of 10%, the average local tax competition increases by 1%, and the average level of economic development quality of neighboring areas increases by 0.042%. At the significance level of 1%, the economic development quality of neighboring regions decreases by 0.052% on average when the local environmental regulation intensity increases by 1%. Compared with the result of point estimation, the impact of tax competition and environmental regulation on the quality of economic development is consistent insignificance and direction, and the size of the coefficients decreases, which supports that it is not accurate to directly use point estimation coefficient to analyze the influence of explanatory variables of the spatial econometric model on the explained variables.

**Table 5:** Direct effect, indirect effect (spatial spillover effect) and total utility of spatial Durbin model.

**Variable**	**Direct effect**		**Indirect effect**		**The total effect**	
	**The coefficient of**	**Robust standard error**	**The coefficient of**	**Robust standard error**	**The coefficient of**	**Robust standard error**
ln*tax*	−0.069***	(0.018)	0.042*	(0.023)	−0.027*	(0.015)
ln*ere*	0.081***	(0.021)	−0.052**	(0.024)	0.029*	(0.016)

*, **, and ***represent the significance level of 10, 5, and 1%, respectively.

### Endogeneity and instrumental variable

The intensity of environmental regulation affects the quality of economic development, and the quality of economic development also affects the intensity of environmental regulation. Using the amount of pollution as the proxy for the intensity of environmental regulation will produce endogenous problems, resulting in biased model estimation results. China mainly promulgates environmental protection laws and regulations, formulating protection regulations, issuing administrative orders on energy conservation and emission reduction and other means to achieve environmental regulation. The government work report is a planning and guiding policy document for economic development and environmental regulation. The willingness and strength of local governments to regulate and improve the environment are fully reflected in the government work report. Based on this, used the ideas of Chen et al. to collect government work reports of 31 provinces in China from 2007 to 2017 (39). Afterword segmentation, the ratio of related environmental words to total words in government work reports was used as the instrumental variable of environmental regulation. This paper selects 13 environment-related words. Compared with Chen et al., who only used 5 environment-related words, the instrumental variables constructed in this paper can more comprehensively reflect the intensity of environmental regulation.

The environmental regulation instrumental variables constructed above can alleviate the endogeneity problem well, but it also implicitly assumes that the willingness and implementation intensity of provincial prefecture-level municipal governments in environmental governance are homogeneous, that is, it cannot effectively reflect the heterogeneity of environmental regulation of provincial prefecture-level municipal governments, which is inconsistent with reality. Since the industry is the main source of pollution in economic development and the proportion of industry in prefecture-level cities is different, the influence of environmental regulation planning and guiding policy documents of provincial governments on their environmental governance efforts and governance intentions will be different. In this paper, the ratio of the total industrial output value of prefecture-level cities to the total industrial output value of the whole province is innovatively adopted to reflect the heterogeneity of the willingness and intensity of environmental regulation of the prefecture-level city governments in the province, and then multiplied by the environmental regulation instrumental variables constructed above to construct heterogeneity of environmental regulation instrumental variables of prefecture-level cities. The instrumental variables constructed in this paper do not directly affect the explained variable (green total factor productivity)^3^, but are highly correlated with the endogenous variable (environmental regulation), satisfying the exogenous hypothesis of the instrumental variables.

Column (1) of Table 6 shows the results based on the method of instrumental variables. In terms of direction and significance, the impact of tax competition and environmental regulation on the quality level of economic development is consistent with the result in Column (2) of Table 4. In terms of direct effect, the direct effect of environmental regulation increases by 13.6%, while the direct effect of tax competition decreases by 43.5%. From the perspective of the spatial spillover effect, the spatial spillover effect of environmental regulation increased by 21.2%, and the spatial spillover effect of tax competition decreased by 30.9%. The results indicate that endogenous problems lead to underestimating the positive effect of environmental regulation policies on promoting Hi-Q ED by local governments, and overestimating the negative effect of inhibiting Hi-Q ED by tax competition.

**Table 6:** Results of instrumental variable and heterogeneous regression.

**Variable**	**Instrumental variable**	**City of heterogeneous**		**Period of heterogeneous**	
	**(1)**	**(2) Large and medium–sized cities**	**(3) Small cities**	**(4) 2007–011**	**(5) 2012–2017**
ln*tax*	−0.046***	−0.043***	−0.051***	−0.048***	−0.043***
	(0.012)	(0.014)	(0.016)	(0.013)	(0.015)
ln*ere*	0.112***	0.102***	0.124***	0.095***	0.126***
	(0.028)	(0.022)	(0.032)	(0.031)	(0.029)
ln*tax*×ln*ere*	0.076*	0.066	0.081**	0.056	0.088**
	(0.044)	(0.043)	(0.040)	(0.043)	(0.041)
***W***×ln*tax*	0.043***	0.057*	0.091***	0.078***	0.039*
	(0.011)	(0.032)	(0.024)	(0.022)	(0.021)
***W***×ln*ere*	−0.074***	−0.046*	−0.094***	−0.076***	−0.053**
	(0.022)	(0.025)	(0.028)	(0.021)	(0.024)
***W***×ln*gtfp*	0.456***	0.384***	0.458***	0.363***	0.487***
	(0.126)	(0.127)	(0.122)	(0.121)	(0.127)
**Direct effcts**					
ln*tax*	−0.039**	−0.036*	−0.047***	−0.043**	−0.38*
	(0.018)	(0.020)	(0.016)	(0.021)	(0.022)
ln*ere*	0.092***	0.082**	0.104***	0.085**	0.116***
	(0.028)	(0.039)	(0.032)	(0.042)	(0.035)
**Indirect or spatial spillover effects**					
ln*tax*	0.029**	0.021*	0.037**	0.032**	0.021*
	(0.014)	(0.012)	(0.018)	(0.015)	(0.011)
ln*ere*	−0.063***	−0.054**	−0.076***	−0.073***	−0.033**
	(0.021)	(0.026)	(0.027)	(0.022)	(0.016)

*, **, and ***represent the significance level of 10, 5, and 1%, respectively. The values in brackets are the corresponding robust standard errors.

### The heterogeneity of city size and period

Economic development and environmental regulation policies are heterogeneous in different city sizes or periods. It is further explored to analyze whether the heterogeneity of city sizes or periods leads to different influences of local government tax and environmental regulation policies on the quality of economic development. Urban size heterogeneity regression results based on environmental regulation instrumental variables are reported in the column (2) and (3) of Table 6, which found that the direct effects of the large and medium-sized cities' tax competition and environmental regulation on the quality of the local economic development are significantly lower than the small cities. That is to say, the improvement of environmental regulation by 1 unit has a more significant effect on improving the quality of economic development in small cities. Under the assumption of the same willingness for environmental regulation, pollution control in small cities is more direct and effective than that in large and medium-sized cities. Therefore, the same environmental regulation policy will be more effective in improving the quality of economic development in small cities. The absolute value of spatial spillover effects of environmental regulation and tax competition in large and medium-sized cities is smaller than that in small cities, indicating that tax competition and environmental regulation policies in small cities are more externalities and spatial exemplary than those in large cities.

Columns (4) and (5) of Table 6 report the model estimation results when the core explanatory variables are examined in the heterogeneous period. It is found that since the 18th CPC National Congress, the direct effect of tax competition on the quality of economic development has not changed significantly. On the contrary, the direct effect of environmental regulation on the quality of economic development has increased significantly, with an average increase of 36.5%. The spatial spillover effect of tax competition and environmental regulation on Hi-Q ED has decreased significantly. Since the 18th National Congress of the Communist Party of China, the intensity of environmental regulations has been continuously enhanced. The government has introduced administrative measures such as interviewing local principals when environmental regulations are weak, and even holding local government principals accountable when environmental regulations are wrong, forcing local governments to improve their efficiency and willingness of implementing environmental policies. The synergistic effect of environmental regulation and tax competition on Hi-Q ED changes from insignificant to significantly positive at the significance level of 5%. It shows that with the improvement of environmental regulation, local governments have been guided to avoid the trap of exchanging the environment for economic growth and turn to high-quality development mode, showing a new trend of coordinated high-quality development of a regional economy.

### Robustness test

To ensure the comparability of the model's estimated results and the reliability of the conclusion, cities above the prefecture-level level were excluded from the sample data, and regression results based on instrumental variables were reported in column (1) of Table 7. The results show that the size and direction of the direct effects of tax competition and environmental regulation on the quality of local economic development are consistent with the previous estimates, which enhances the spatial spillover effect of tax competition and environmental regulation on the quality of economic development, but the change is not significant. In order to avoid the impact of outliers in the sample data on the regression results, the sample data of the lowest and highest 0.5% in the samples of environmental regulation and tax competition were further excluded. The estimated results after the exclusion of outliers were reported in column (2) of Table 7. Although the direct effect and spatial spillover effect of environmental regulation and tax competition on the quality of economic development decreased, the direction and significance level did not change, indicating that the outliers had no significant impact on the model regression results. Based on the Bootstrap method (self-sampling method), regression was performed on the model to test the sensitivity of the conclusion to the data. Col. (3) of Table 7 is the estimation result of the self-sampling 1,000 times. It can be seen that the direct effect and spatial spillover effect of tax competition and environmental regulation on Hi-Q ED do not change significantly. Therefore, the direct effect and spatial spillover effect of the spatial Durbin model based on instrumental variable estimation described above are robust.

**Table 7:** The results of the Robustness test.

**Variable**	**Spatial weight of economic distance**			**Spatial weight of adjacent**			**Spatial weight of economic distance**		
	**Prefecture-level cities**	**Exclude outliers**	**Bootstrap**	**Prefecture-level cities**	**Exclude outliers**	**Bootstrap**	**Prefecture-level cities**	**Exclude outliers**	**Bootstrap**
	**(1)**	**(2)**	**(3)**	**(4)**	**(5)**	**(6)**	**(7)**	**(8)**	**(9)**
ln*tax*	−0.071***	−0.061***	−0.064***	−0.072***	−0.060***	−0.069***	−0.065***	−0.078***	−0.067***
	(0.019)	(0.015)	(0.017)	(0.023)	(0.020)	(0.023)	(0.021)	(0.025)	(0.022)
ln*ere*	0.118***	0.108***	0.106***	0.102***	0.109***	0.112***	0.120***	0.107***	0.119***
	(0.024)	(0.023)	(0.021)	(0.019)	(0.031)	(0.023)	(0.035)	(0.029)	(0.032)
ln*tax*×*ere*	0.067*	0.056*	0.059*	0.066**	0.053*	0.062*	0.072**	0.068**	0.056*
	(0.038)	(0.031)	(0.033)	(0.032)	(0.030)	(0.035)	(0.035)	(0.032)	(0.030)
***W***×ln*tax*	0.085***	0.076***	0.071***	0.071***	0.087**	0.069**	0.069***	0.076**	0.081**
	(0.023)	(0.022)	(0.021)	(0.023)	(0.034)	(0.026)	(0.023)	(0.028)	(0.036)
***W***×ln*ere*	−0.084***	−0.072***	−0.083***	−0.079***	−0.062**	−0.065**	−0.060**	−0.082***	−0.056**
	(0.023)	(0.021)	(0.026)	(0.023)	(0.030)	(0.029)	(0.023)	(0.025)	(0.022)
***W***×ln*gftp*	0.689***	0.554***	0.653***	0.665***	0.651***	0.507***	0.501***	0.605***	0.632***
	(0.126)	(0.136)	(0.127)	(0.132)	(0.129)	(0.128)	(0.121)	(0.127)	(0.139)
**Direc effects**									
ln*tax*	−0.041**	–0.036**	−0.034**	−0.046**	−0.040**	−0.044**	−0.056**	−0.050**	−0.054**
	(0.020)	*(0.017)*	(0.016)	(0.022)	(0.020)	(0.021)	(0.027)	(0.024)	(0.026)
ln*ere*	0.109***	0.095***	0.107***	0.108***	0.099***	0.102***	0.118***	0.109***	0.112***
	(0.035)	*(0.032)*	(0.031)	(0.034)	(0.032)	(0.033)	(0.036)	(0.033)	(0.035)
**Indirect or spatial spillover effects**									
ln*tax*	0.032**	0.026*	0.027**	0.031**	0.029*	0.036**	0.041**	0.034*	0.046**
	(0.016)	(0.015)	(0.012)	(0.014)	(0.017)	(0.018)	(0.020)	(0.018)	(0.022)
ln*ere*	−0.071***	−0.061***	−0.068***	−0.073***	−0.062***	−0.065***	−0.081***	−0.082***	−0.078***
	(0.024)	(0.020)	(0.023)	(0.025)	(0.020)	(0.021)	(0.026)	(0.027)	(0.024)

***, ** and *represent the significance level of 1, 5 and 10%, respectively. The values in brackets are the corresponding robust standard errors.

The above robustness test is based on the spatial weight of geographical distance. In order to avoid the error of estimation result caused by the improper selection of spatial weight, columns (4)–(6) and (7)–(9) of Table 7 are the results of re-estimation^4^ of the above robustness test method based on the weight of adjacent space and spatial weight of economic distance. Compared with the estimation results based on geographical distance and spatial weight in columns (1)–(3), it is found that the significance level, direction and magnitude of correlation effects of tax competition and environmental regulation do not change significantly. This indicates that spatial weight does not lead to bias in model estimation, and the results of spatial weight estimation based on geographical distance are robust.

## Conclusions

Studying economic development in China is a very important topic recently because China's economy is moving toward high-quality development, and local governments face the dilemma of environmental governance and economic development. To contribute to the literature in this area further, this paper empirically examines the impacts of both environmental governance and tax competition on economic development quality by using a constructed spatial econometric model and by using data from 278 cities at the prefecture level and above in China from 2007 to 2017. The empirical results obtained in our paper show that tax competition inhibits the high-quality development of the local economy and has a positive spatial spillover effect, while environmental governance has a significant direct promotion and a negative spatial spillover effect on the high-quality development of the local economy. Furthermore, we found that local government tax competition inhibits the promotion effect of environmental governance on high-quality economic development, and there are both regional heterogeneity and time heterogeneity in the impact of different city sizes and periods of implementing tax competition and environmental governance policies on the quality of economic development. Finally, we find that the direct effects and spatial spillover effects of tax competition and environmental governance on the quality of economic development in large and medium-sized cities are significantly lower than those in small cities. Since the 18th National Congress of the Communist Party of China, the improvement effect of environmental governance on the quality of economic development and its synergistic effect with tax competition have become more and more significant.

In order to realize the development vision of a new era that requires both “gold and silver mountains” and “clear water and green mountains”, the CPC Central Committee and local governments need to further adjust their strategies and optimize relevant policies. One is to weaken the economic assessment indicators for local officials' promotion. In the new era, we will implement the new concept of officials' promotion and evaluation, guide the transformation of economic competition between local governments, enhance the motivation of local officials to develop a high-quality economy, and meet the growing needs of the people for the “two mountains”. Therefore, in the assessment of official performance, the “hero” assessment method based on GDP should no longer be adopted, and a diversified evaluation system including environmental weight should be improved to form a new era of evaluation system of official performance. Secondly, the findings in our paper can be used to guide green tax competition among local governments. Tax competition leads to administrative forces interfering in the market's function of resource allocation, causing local governments to promote GDP growth while causing problems such as resource mismatch and environmental deterioration. The central government should further regulate the tax competition behavior among local governments, regulate their jurisdiction, reduce the local tax preferential behavior that is not conducive to environmental improvement, and guide the tax competition among local governments to change from the bottom competition to the top competition. The third is to strengthen the pushback effect of environmental regulation. We correct the mismatch of production factors through environmental regulation, put in place a mechanism to guide the allocation of market factors through environmental regulation, create conditions for the development of new drivers, and force the green upgrading of industries. Fourth, we improve the coordination mechanism for high-quality regional economic development. Local policies to promote high-quality economic development may not have any significant effect after implementation due to the spatial interactivity of the policies themselves. In order to realize high-quality economic development, it is necessary to guide local governments to plan and implement the joint supervision, monitoring and early warning system of high-quality economic development, and further improve the collaborative mechanism of regional high-quality economic development.

Since the work reports of prefecture-level cities are generally prepared according to the provincial work reports, and many work reports of prefecture-level cities are not published publicly, especially before 2012. In this research, when constructing the instrumental variables of environmental governance in prefecture-level cities, we use the provincial government work report to extract certain vocabulary for construction, instead of directly using the prefecture-level municipal government work report. Although it solves the endogenous problem well, it also has certain limitations. Next, we collect the work reports of prefecture-level municipal governments after 2012 to further explore the endogenous problems of environmental governance. The findings in our paper are useful for both the central government and the local governments in making better decisions for economic development in China as well as in other countries. In this paper, we apply the spatial Durbin model, instrumental variable, and heterogeneity analysis to assess the impact of tax competition and environmental regulation on high-quality economic development. Extensions of our paper include applying our approach to study other development issues (41–48), policy issues (49–51), economic and financial risks (52–55), and many other important issues.

## Data availability statement

Publicly available datasets were analyzed in this study. This data can be found here: The data are from China City Statistical Yearbook.

## Author contributions

All authors listed have made a substantial, direct, and intellectual contribution to the work and approved it for publication.

## Funding

Thanks for the support of the 2022 Henan Philosophy and Social Science Planning Project-Research on the Government Empowerment Mechanism for Improving the Ability of Poverty Alleviation Farmers to Build Assets (grant number: 2022BJJ089), the 2022 Henan Soft Science Research Project-Research on the Path of Improving Henan's Total Factor Productivity Under High-Quality Economic Development (grant number: 222400410087), and the 2021 Henan New Liberal Arts Research and Reform Practice Project-Innovation and Practice Research on the New Business Talent Training System of Local Colleges and Universities Under the Empowerment of Information Technology (grant number: JG [2021] No. 175). In addition, this research has been supported by Xinyang Normal University, Yancheng Institute of Technology, Guizhou University of Finance and Economics, Asia University, China Medical University Hospital, The Hang Seng University of Hong Kong, Research Grants Council (RGC) of Hong Kong (project numbers 12502814 and 12500915), and the Ministry of Science and Technology (MOST, Project Numbers 106-2410-H-468-002 and 107-2410-H-468-002-MY3), Taiwan.

## Conflict of interest

The authors declare that the research was conducted in the absence of any commercial or financial relationships that could be construed as a potential conflict of interest.

## Publisher's note

All claims expressed in this article are solely those of the authors and do not necessarily represent those of their affiliated organizations, or those of the publisher, the editors and the reviewers. Any product that may be evaluated in this article, or claim that may be made by its manufacturer, is not guaranteed or endorsed by the publisher.

## Footnotes

^1^The logic of using the spatial weight of geographical distance is that this paper discussed tax competition and environmental regulation is more of a policy of interactions between adjacent areas or imitation, it is calculated according to the city of latitude and longitude geographic distance between cities, and then take its inverse, the greater the distance between each other, giving you less weight, the influence of and vice versa. In order to reduce or eliminate external influences between regions and make W dimensionless, the weight matrix is normalized to have the sum of the row elements equal to 1.

^2^In order to avoid serious multicollinearity problems leading to deviation of the estimated results, the variance inflation factor (VIF) test was carried out in this paper before regression, and its value was 6.8, which showed no serious multiple common problems.

^3^In the actual operation process, Anderson Canon LM test, Cragg-Donald *F* test and Sargan-Hansen test are adopted in this paper to test the constructed instrumental variables. It shows that the instrumental variables have problems such as insufficient recognition, weak recognition and excessive recognition. In order to maintain the coherence of the paper, no longer report on inspection procedures.

^4^Anselin (40) pointed out that there are generally two kinds of adjacent weights in the construction space: Rook proximity and Queen proximity, in which the latter proximity refers to the fact that there is only a common boundary, and the latter proximity also includes a common vertex in addition to the common boundary (40). In this paper, the weight of the post-adjacent space is adopted. In addition, in addition to the first-order adjacent matrix, the weight of the adjacent space also includes the neighboring matrix of higher order. The spatial adjacent weight in this paper only considers the post-first-order adjacent situation.
